# Orbital hybridization-mediated synergistic multi-electron redox in a NASICON cathode unlocking solid-solution reactions for ultrafast and durable sodium storage

**DOI:** 10.1039/d6sc01289b

**Published:** 2026-03-23

**Authors:** Yi-Fei Liu, Jin-Ling Liu, Xiao-Tong Wang, Yan Zhuang, Jin-Zhi Guo, Heng Zhang, Denglong Chen, Zhen-Yi Gu, Xing-Long Wu

**Affiliations:** a College of Environmental and Resource Sciences, College of Carbon Neutral Modern Industry, Fujian Normal University Fuzhou 350007 P. R. China dlchen@fjnu.edu.cn; b State Key Laboratory of Integrated Optoelectronics, MOE Key Laboratory for UV Light-Emitting Materials and Technology, School of Physics, Northeast Normal University Changchun Jilin 130024 P. R. China guzy166@nenu.edu.cn xinglong@nenu.edu.cn; c Quangang Petrochemical Research Institute, Fujian Normal University Quanzhou 362801 P. R. China

## Abstract

Na_4_VMn(PO_4_)_3_, as a high-energy-density and low-cost cathode material for sodium-ion batteries (SIBs), holds promising application prospects. However, its practical performance is limited by the stepwise redox reactions of V and Mn, which induce significant phase transitions and sluggish kinetics, particularly during the second desodiation process. To address this issue, we propose an orbital hybridization regulation strategy based on Ti/Fe co-doping. By tailoring the local coordination environment, the introduced Ti/Fe constructs a 3d–3d metallic network, inducing continuous multi-orbital hybridization. This transforms the V/Mn redox process from stepwise to simultaneous, eliminating sharp phase boundaries and overcoming the kinetic bottleneck in the second desodiation step. Additionally, the d-band energy level difference between V and Mn is narrowed to 0.701 eV, enhancing electron delocalization and intrinsic conductivity, thereby enabling highly reversible multi-electron transfer processes. The optimized Na_3.75_V_0.75_Mn_0.75_Ti_0.25_Fe_0.25_(PO_4_)_3_ effectively mitigates volumetric stress and local phase transitions, ensuring structural integrity. Consequently, the material retains 73% capacity after 2000 cycles at 10C, demonstrating superior rate capability and cycling stability. This work provides crucial electronic-level insights and a novel design paradigm for high-performance SIB cathode materials.

## Abstract 2

Ti/Fe co-doping modulates the electronic structure of NVMTFP, enabling single-step desodiation. It reduces the Na+ migration energy barrier, accelerates ion diffusion, and alleviates lattice distortion.

## Graphical abstract

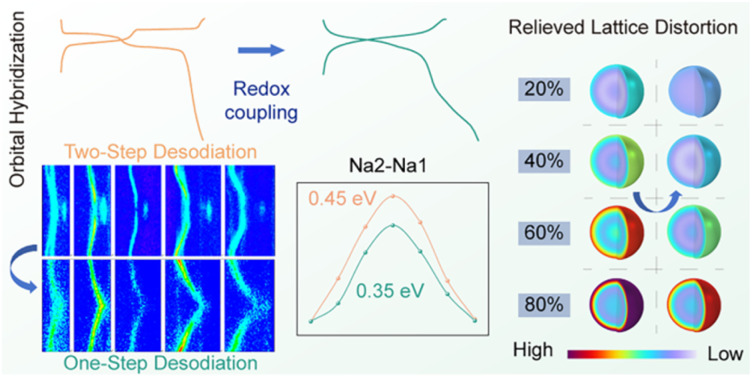

## Introduction

With the global escalating demand for grid integration of renewable energy and smart grid storage, developing novel electrochemical energy storage systems that surpass existing lithium-ion battery technology—featuring low cost, high safety, and long lifespan—has become an urgent priority.^1–6^ Sodium-ion batteries (SIBs), recognized for their core advantages including abundant elemental reserves, cost-effectiveness, and analogous working mechanisms to lithium-ion batteries, are widely regarded as highly promising candidates for large-scale energy storage applications.^7–11^ Against this backdrop, cathode materials, as critical components determining battery energy density, power density, and cycling stability, represent a central challenge in advancing SIB technology. Among diverse SIB cathode systems, sodium super ionic conductor (NASICON)-type polyanionic compounds, particularly Na_4_VMn(PO_4_)_3_ (NVMP), have garnered significant attention due to their robust open three-dimensional framework, high operating voltage, and exceptional thermal stability.^12,13^ Compared with sodium-ion positive electrode materials such as Na_3_V_2_(PO_4_)_3_,^14^ Na_4_Fe_3_(PO_4_)_2_P_2_O_7_,^15,16^ and NaFePO_4_,^17^ NVMP can activate both V^3+^/V^4+^ and Mn^2+^/Mn^3+^ redox couples, thereby offering a theoretical specific capacity 111 mAh g^−1^, and it has more significant advantages in terms of magnification performance and energy density.^18–20^

However, its practical electrochemical performance is severely constrained by inherent kinetic and structural instabilities associated with the multi-step desodiation mechanism. Specifically, within the voltage window of 1.8–3.8 V (*vs.* Na^+^/Na), NVMP exhibits two distinct voltage plateaus at 3.4 V and 3.6 V, corresponding to sequential redox reactions of V and Mn, respectively. Research indicates severe kinetic limitations during the second desodiation step (Mn^2+^/Mn^3+^); one-dimensional Na^+^ diffusion along the *c*-axis is impeded by preferential vacancy formation at Na(2) sites and occupation-induced blocking at Na(1) sites, resulting in a diffusion coefficient one order of magnitude lower than that of the first step, critically limiting rate capability.^21^ More challengingly, the inherent Jahn–Teller effect of Mn^3+^ induces pronounced lattice distortion, generating cumulative structural strain during repeated charge–discharge cycles, ultimately leading to particle fracture and rapid capacity decay. Consequently, the performance bottleneck of the NVMP material fundamentally stems from the combined effects of heterogeneous de-/sodiation kinetics and phase transition-induced structural stress.^22,23^

Transforming the traditional multi-step two-phase transition reactions into a single solid-solution reaction represents an ideal pathway to address the aforementioned challenges.^24,25^ The solid-solution reaction mechanism proceeds in a single-phase, continuous manner with minimal volume variation, effectively mitigating internal stress accumulation and interfacial degradation caused by repeated phase transitions, thereby significantly improving the cycling durability of the material.^26^ The key to achieving this transformation lies in the precise regulation of the local electronic structure of the material.^27,28^ Orbital hybridization, as a powerful tool for electronic structure engineering, offers a novel perspective for this purpose. By introducing appropriate heterogeneous elements into the NASICON structure, orbital reconstruction of metal–oxygen bonds can be induced, strengthening their covalent character.^29,30^ This enhanced covalency not only facilitates the construction of a continuous electron transport network, improving intrinsic electrical conductivity, but also effectively “locks” the coordination environment of transition metal ions, suppressing Jahn–Teller distortions and smoothening the energy barrier during the de-/sodiation process.^31–33^

Consequently, these effects create essential conditions for realizing highly reversible solid-solution reactions. Despite its promising prospects, precisely steering the reaction pathway toward a solid-solution mechanism through synergistic multi-element design—while simultaneously activating multi-electron redox processes—remains a significant challenge. Based on this, our study proposes an orbital hybridization-mediated cooperative doping strategy. We designed and synthesized a novel Na_3.75_V_0.75_Mn_0.75_Ti_0.25_Fe_0.25_(PO_4_)_3_ (NVMTFP) cathode material, aiming to restructure the electronic structure through the synergistic introduction of Ti and Fe. Comprehensive electrochemical tests, *in situ* X-ray diffraction (XRD), galvanostatic intermittent titration technique (GITT), and density functional theory (DFT) calculations collectively confirmed that Ti/Fe co-doping successfully induced a strong orbital hybridization effect. This not only significantly enhanced the Na^+^ diffusion kinetics but, more importantly, fused the originally separated redox peaks of V^3+^/V^4+^ and Mn^2+^/Mn^3+^, transforming the entire charge–discharge process into a highly reversible solid-solution reaction mechanism. Consequently, the NVMTFP material exhibits outstanding cycling stability, retaining over 73% of its initial capacity after 2000 cycles at 10C in half cells. Even in full cells, it retains 89% of its capacity after 200 cycles at 2C. This work elucidates, at the electronic level, the regulatory mechanism of orbital hybridization on the reaction pathway, providing innovative design principles and robust theoretical foundations for developing next-generation high-performance, durable cathode materials for SIBs.

## Results and discussion

**Fig. 1:**
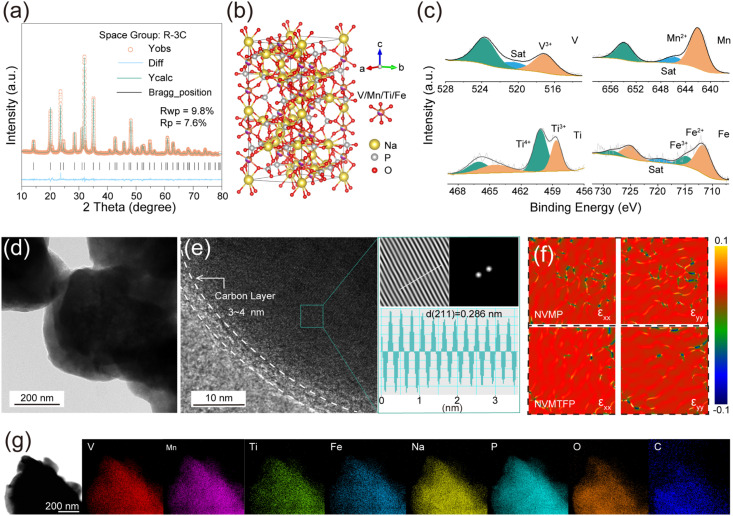
(a) XRD Rietveld refinement results of NVMTFP. (b) Schematic crystal structure of NVMTFP. (c) High-resolution V 2p, Mn 2p, Ti 2p and Fe 2p XPS spectra of NVMTFP. (d) TEM image of NVMTFP. (e) HRTEM image with the corresponding FFT (top right) and line profile (bottom right). (f) Localized strain distribution analysis by GPA. (g) EDS mapping of NVMTFP.

To investigate the impact of Ti/Fe co-doping on the crystal structure of the material, we performed XRD analysis and Rietveld refinement on both the NVMTFP and NVMP samples. As shown in Fig. 1a and S1, the diffraction patterns of NVMTFP and NVMP exhibit high consistency, with all characteristic peaks being indexed to the rhombohedral crystal system with space group *R*3̄*c*, indicating that the doping process did not alter the primary framework structure of the material.^34^ The refinement results (Tables S1–S3) further confirm that Ti^4+^ and Fe^2+^ were successfully incorporated into the transition metal (TM) sites, occupying the 12c Wyckoff position. Due to minor ionic radius discrepancies between Fe^2+^ (∼0.78 Å) and the native Mn^2+^ cation (∼0.835 Å) and a close radius match between V^3+^ (∼0.64 Å) and Ti^4+^ (∼0.605 Å), their substitution induced minor contraction in lattice parameters *a* and *b*, leading to a reduction in unit cell volume.^35^ This subtle lattice shrinkage serves as direct structural evidence for successful dopant incorporation and its influence on the local bonding environment. Fig. 1b illustrates the crystal structure model of NVMTFP. Its framework is constructed by corner-sharing connections between [MO_6_] octahedra formed by TM and oxygen atoms, along with [PO_4_] tetrahedra, forming a unique lantern-like three-dimensional open network. This architecture provides continuous and spacious diffusion pathways for de-/sodiation, laying the structural foundation for the favorable ionic conductivity of the material. Additionally, Fourier-transform infrared spectroscopy (FT-IR) analysis corroborates the presence of local chemical bonds (Fig. S2).^36^ Distinct absorption peaks corresponding to stretching vibrations of TM–O and P–O bonds were clearly observed, further validating the existence and chemical integrity of the fundamental building units ([MO_6_] octahedra and [PO_4_] tetrahedra) within the materials. Furthermore, X-ray photoelectron spectroscopy (XPS) was employed to characterize the surface composition and elemental valence states. As shown in Fig. 1c, the V 2p spectrum reveals that the binding energies of V 2p_3/2_ and V 2p_1/2_ are 517.1 eV and 523.8 eV, respectively, which are consistent with V^3+^ reported in the literature.^36^ In the Mn 2p spectrum, characteristic peaks at 641.8 eV and 653.6 eV correspond to Mn^2+^, with a distinct satellite peak detected at 646.9 eV.^37^ Further analysis of the valence states of Ti and Fe shows that both elements exhibit mixed valence states in their spectra. In the Ti 2p spectrum, characteristic peaks at 458.6 eV and 464.5 eV are attributed to Ti^3+^, while those at 460.1 eV and 466.1 eV belong to Ti^4+^.^38^ Similarly, the characteristic peaks at 711.6 eV and 725.05 eV correspond to Fe^2+^, while those at 714.65 eV and 728.45 eV are assigned to Fe^3+^ in the Fe 2p spectrum.^39^ XPS survey spectra (Fig. S3) reveal the full elemental composition of NVMP and NVMTFP, and the XPS spectra of V and Mn in NVMP exhibit characteristic binding energy peaks similar to those in NVMTFP, confirming that the introduction of Ti and Fe does not alter the valence states of V and Mn. Subsequently, transmission electron microscopy (TEM) was employed to characterize the morphology of the material, and the TEM image of NVMTFP (Fig. 1d) reveals that it is composed of irregular particles. The high-resolution TEM (HRTEM) image of NVMTFP (Fig. 1e) manifests distinct lattice fringes with an interplanar spacing of 0.286 nm, corresponding to the (211) crystal plane.

Additionally, the particles are coated with a continuous, uniform carbon layer. Moreover, this carbon layer not only enhances the surface electronic conductivity but also effectively mitigates electrolyte corrosion, thereby improving the cycling stability.^40,41^ Notably, geometric phase analysis (GPA) demonstrates that such co-doping markedly alleviates the lattice strain of the material (Fig. 1f).^42^ Specifically, NVMP exhibits marked heterogeneity in strain distribution with numerous extreme strain domains. Furthermore, the thermogravimetric (TG) analysis (Fig. S4) and Raman spectroscopy (Fig. S5) results reveal that Ti/Fe co-doping exerts no significant influence on the carbon content or the degree of graphitization. As shown in Fig. 1g, energy dispersive spectroscopy (EDS) elemental mapping confirms that all elements (Na, V, Mn, Ti, Fe, P, O, and C) are uniformly distributed across the particles, which further verifies the successful doping of Ti and Fe into the lattice.

**Fig. 2:**
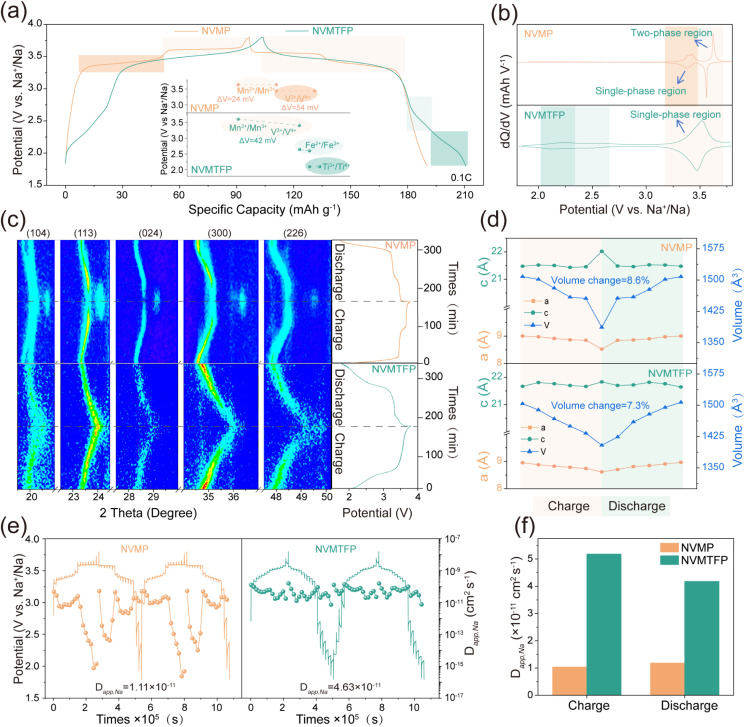
(a) The comparison of GCD curves at 0.1C. (b) The d*Q*/d*V* curves. (c) Contour maps of *in situ* XRD and corresponding GCD curves. (d) Corresponding unit cell volume variation. (e) The GITT curves and calculated *D*_app,Na_. (f) The comparison of *D*_app,Na_ at different states.

The electrochemical impact of the orbital hybridization-mediated strategy was evaluated by analyzing the galvanostatic charge–discharge (GCD) curves and corresponding d*Q*/d*V* curves in half-cells. As depicted in Fig. 2a, during the charge–discharge process, NVMP exhibits two distinct voltage plateaus above 3 V, corresponding to the redox couples of V^3+^/V^4+^ and Mn^2+^/Mn^3+^ with polarization voltages of 24 mV and 54 mV, respectively, and a discharge specific capacity of 95.53 mAh g^−1^. Notably, NVMTFP exhibits remarkably distinct electrochemical behavior. The two previously separate plateaus merge into a single sloped voltage profile. This profile features a polarization voltage of 42 mV, while the discharge specific capacity of NVMTFP is enhanced to 102.68 mAh g^−1^. The d*Q*/d*V* curves in Fig. 2b further confirm the intrinsic differences between the two materials in their redox processes. For NVMP, two distinct redox peaks are observed, corresponding to the single-phase V^3+^/V^4+^ and two-phase Mn^2+^/Mn^3+^ redox couples.^20^ In contrast, NVMTFP shows three redox peak pairs at about 2.15, 2.56, and 3.52 V, which are attributed to the Ti^3+^/Ti^4+^, Fe^2+^/Fe^3+^, and the coupled V^3+^/V^4+^ – Mn^2+^/Mn^3+^ redox reactions, respectively. These results confirm that adopting orbital hybridization-mediated synergistic doping effectively promotes the coupling of V/Mn redox reactions and homogenizes the polarization between the original dual plateaus.

*In-situ* XRD was used to investigate the Na^+^ storage mechanism and structural evolution of NVMP and NVMTFP electrodes during the de-/sodiation process. As illustrated in Fig. 2c and S6, the NVMP and NVMTFP electrodes exhibit distinct Na^+^ storage mechanisms. During the voltage plateau at ∼3.4 V, only a continuous shift of diffraction peaks is observed without the emergence of new peaks. This indicates that a single-phase reaction occurs in NVMP during the process. However, distinct decreases are observed in the diffraction peak intensities corresponding to the (104), (113), (024), (300), and (226) crystal planes at ∼3.6 V, accompanied by the emergence of new diffraction peaks, which is characteristic of a typical two-phase reaction.^43^ In contrast, NVMTFP only exhibits a gradual shift of these diffraction peaks to higher angles without new peaks forming, confirming a solid-solution reaction mechanism throughout the charge–discharge process.^21,44^ After the discharge is complete, all diffraction peaks of both electrodes revert to their initial positions, demonstrating excellent reversibility of de-/sodiation. The evolution of unit cell parameters during cycling is shown in Fig. 2d. NVMTFP displays a smooth volume variation with a total change of only 7.33%, whereas NVMP undergoes an abrupt phase transition with a volume change of 8.6%. These results confirm that Ti/Fe co-doping at the TM sites modulates the electron-filling state, promoting the coupling of the V/Mn redox plateaus. This coupling enables a one-step desodiation process, thereby alleviating the risk of irreversible phase transitions during de-/sodiation.^35^

Meanwhile, the Na^+^ diffusion kinetics was assessed using the GITT. The linear relationship between the peak current and the square root of the scan rate for single titration measurements (Fig. S7 and S8) confirms the diffusion-controlled de-/sodiation behavior.^45^ The GITT profiles of NVMP and NVMTFP and the corresponding Na^+^ diffusion coefficients (*D*_app,Na_) reveal distinct kinetic behaviors in the operating voltage range (Fig. 2e). In NVMP, the redox process of V^3+^/V^4+^ at 3.4 V is a single-phase reaction, with the *D*_app,Na_ values remaining stable between 10^−10^ and 10^−11^ cm^2^ s^−1^. However, when the voltage reaches 3.6 V, the *D*_app,Na_ values drop sharply to approximately 10^−15^ cm^2^ s^−1^, showing significant fluctuations. This sudden decrease in the *D*_app,Na_ value is caused by the structural phase transition induced by Mn^2+^/Mn^3+^ redox, which hinders the original migration path and severely limits the Na^+^ diffusion ability.^21^ In contrast, in the NVMTFP cathode with a solid-solution Na^+^ storage mechanism, *D*_app,Na_ remains stable in the range of 10^−11^ to 10^−10^ cm^2^ s^−1^, and the ion migration behavior is remarkably consistent during the charge–discharge process, with no significant diffusion barriers. The average *D*_app,Na_ value over the entire cycle reaches 4.63 × 10^−11^ cm^2^ s^−1^, in contrast to a value of 1.11 × 10^−11^ cm^2^ s^−1^ for NVMP. Across both charging and discharging processes, NVMTFP sustains a substantially higher *D*_app,Na_ value, as presented in Fig. 2f. Collectively, these findings confirm that Ti/Fe co-doping results in effective V/Mn potential coupling, which eliminates the kinetic disparity between the redox couples and enhances the overall Na^+^ diffusion kinetics. This synergistic effect enables a one-step de-/sodiation process, which is key to its excellent electrochemical performance.

**Fig. 3:**
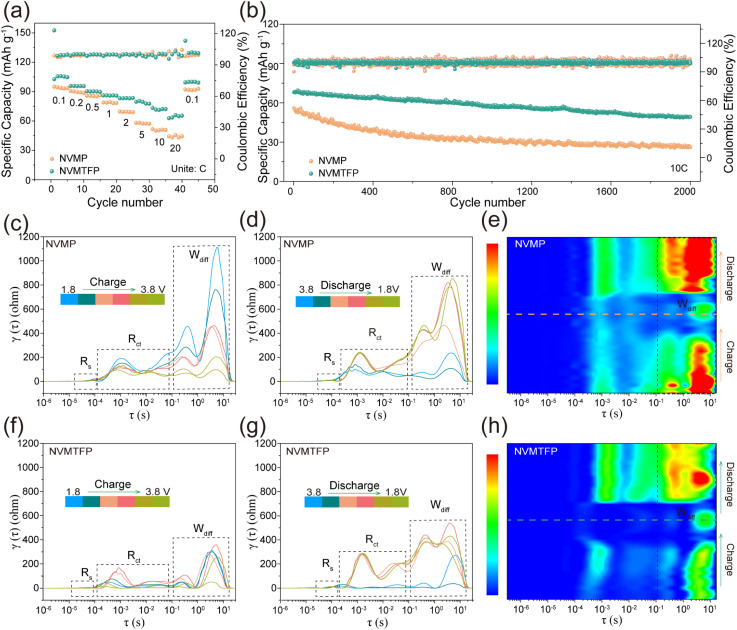
(a) Rate capability at varied current densities. (b) Cycling performance at 10C. (c and d) The DRT curves and (e) corresponding contour maps of NVMP during the redox process. (f and g) The DRT curves and (h) corresponding contour maps of NVMTFP during the redox process.

The NVMTFP and NVMP half-cells were tested at a continuously varying rate from 0.1 to 20C to further analyze the impact of the orbital hybridization-mediated strategy on their electrochemical properties. As shown in Fig. 3a, NVMTFP exhibits superior discharge specific capacity at all current densities. Specifically, it delivers discharge specific capacities of 102.68, 95.76, 90.28, 86.13, 83.21, 79.62, and 73.41 mAh g^−1^ at 0.1, 0.2, 0.5, 1, 2, 5 and 10C, in contrast to NVMP, which exhibits corresponding values of 95.53, 90.79, 83.65, 75.31, 62.32, 52.83, and 49.65 mAh g^−1^. Even at 20C, the discharge specific capacity of NVMTFP remains 62.90 mAh g^−1^, significantly surpassing that of NVMP (44.38 mAh g^−1^). Notably, compared with NVMP, the discharge curves of NVMTFP (Fig. S9) all manifested as smooth sloping profiles at different current densities, indicating a stable solid-solution reaction. Furthermore, NVMTFP demonstrates superior cycling performance. As shown in Fig. S10, after 300 cycles at 1C, the NVMTFP cathode delivers a discharge specific capacity of 78.06 mAh g^−1^ with a capacity retention rate of 89%. In contrast, the NVMP cathode exhibits a considerably lower discharge specific capacity. When the current density reaches as high as 10C, the NVMTFP cathode retains 73% of its capacity after 2000 cycles (Fig. 3b), while the NVMP only retains 46%. By comparing the discharge curves during the cycling process at 1C (Fig. S11a and b), NVMP and NVMTFP both retain their respective intrinsic curve features well. In sharp contrast, at 10C, the capacity degradation of NVMTFP is much lower, and it exhibits better curve retention performance (Fig. S11c and d). Additionally, to accommodate diverse operating environments, the high-low temperature cycling stability of NVMTFP was evaluated. At 50 °C, after 500 cycles at 5C, the capacity retention remained as high as 81%. Even at −40 °C, the capacity retention reaches 78% after 250 cycles at 0.5C (Fig. S12). From the above analysis, Ti/Fe co-doping enhances the diffusion kinetics at the redox plateau, improves structural stability, and ultimately enhances the performance of NVMP.

Additionally, *in situ* electrochemical impedance spectroscopy (EIS) measurements were conducted on both samples, coupled with distribution of relaxation times (DRT) analysis, to enable comprehensive insights into the electrochemical kinetic characteristics across different stages.^46,47^ The Nyquist plots demonstrate that NVMTFP exhibits significantly lower initial resistance (*R*_ct_) and diffusion resistance (*W*_diff_) than NVMP throughout the charging process (Fig. S13). Specifically, the NVMTFP exhibited a smaller impedance semicircle and a steeper low-frequency line at 1.8 V (Fig. S14), which directly proved that the charge transfer and diffusion kinetics of NVMTFP were significantly enhanced. Meanwhile, the corresponding DRT spectrum consistently reveals a low peak value (Fig. 3c and f). During the subsequent discharge process (Fig. 3d and g), the *W*_diff_ value of NVMP increases sharply with voltage variation, while NVMTFP shows no significant fluctuation. As shown in Fig. 3e and h, the disparity in impedance characteristics is further clearly reflected with NVMTFP exhibiting a significantly lower *W*_diff_ value than NVMP. This reflects its superior Na^+^ migration kinetics, which are consistent with the GITT test data. Meanwhile, within the 3.4–3.8 V voltage range, the *R*_ct_ value of NVMTFP is consistently lower than that of NVMP (Fig. S15), which is attributed to the optimized electron density.^30^ Besides, as shown in Fig. S16, within the tested pressure range, the electronic conductivity of NVMTFP was significantly higher, which is consistent with the results of DRT. Therefore, Ti/Fe co-doping significantly enhances electronic conductivity while reducing Na^+^ diffusion resistance, thereby endowing the material with core electrochemical advantages of both exceptional rate capability and stable capacity retention.

**Fig. 4:**
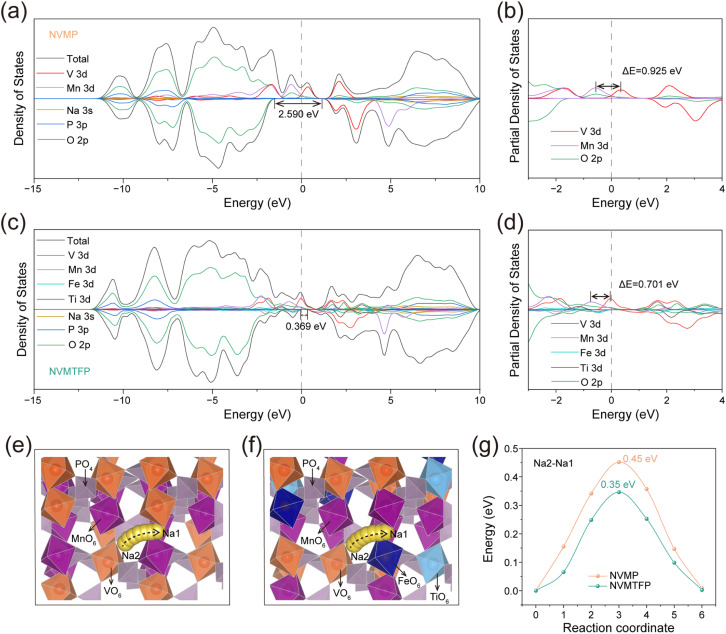
(a) and (c) DOS of the NVMP and NVMTFP samples, respectively. (b) and (d) Corresponding PDOS. (e) and (f) Schematic illustration of Na^+^ migration along the Na2–Na1 pathway in NVMP and NVMTFP. (g) Corresponding Na^+^ migration energy barrier.

DFT calculations were conducted to elucidate the regulatory effect of Ti/Fe co-doping on the electronic structure of NVMP. As depicted in Fig. 4a and c, density of states (DOS) analysis shows that the spin-down state band gap at the Fermi level narrows after Ti/Fe co-doping (from 2.59 to 0.369 eV), indicating that co-doping enhances electronic conductivity and optimizes charge transfer kinetics. Furthermore, within the −4 to 4 eV energy range, the d orbitals introduced by Ti/Fe co-doping overlap with the original d orbitals in NVMP, thereby forming a 3d–3d metallic network, which eliminates the energy band gap and enables continuous orbital hybridization.^48^ Partial density of states (PDOS) analysis (Fig. 4b and d) demonstrates that the peaks of V 3d and Mn 3d near the Fermi level (EF) are separated by 0.925 eV, a separation reflecting weak hybridization with O 2p orbitals and electronic decoupling between the two metal centers. This energy-level distinction induces stepwise electronic activation, with redox reactions proceeding independently at individual V and Mn sites *via* discontinuous electron transfer. This stepwise electronic activation aligns with the experimentally observed stepwise desodiation behavior. For NVMTFP, the peak separation between V and Mn d-bands narrows to 0.701 eV. This reduction signals electronic state unification and a corresponding decrease in the redox potential gap that underpins the transition from stepwise to concurrent redox reactions. Therefore, this multi-orbital synergy enhances electron delocalization to improve intrinsic electronic conductivity and constructs continuous charge transfer pathways, thereby overcoming the limitations of single-metal-center activation. This enables the synchronous and continuous redox reactions of V and Mn, thereby eliminating the thermodynamic driving force for phase separation. This regulatory effect not only optimizes the Na^+^ diffusion channel and eliminates the kinetic bottleneck but also alleviates the Jahn–Teller effect of Mn^3+^ and reduces lattice distortion. Then, the Na^+^ migration energy barrier was calculated *via* DFT to further elucidate the effect of co-doping on Na^+^ transport (Fig. 4e and f).^49^ The calculation results are shown in Fig. 4g; the Na^+^ migration energy barrier of NVMP is 0.45 eV, while that of NVMTFP is significantly reduced to only 0.35 eV. This result clearly demonstrates that the solid solution reaction induced by Ti/Fe co-doping significantly accelerates the Na^+^ transport rate.^50^

**Fig. 5:**
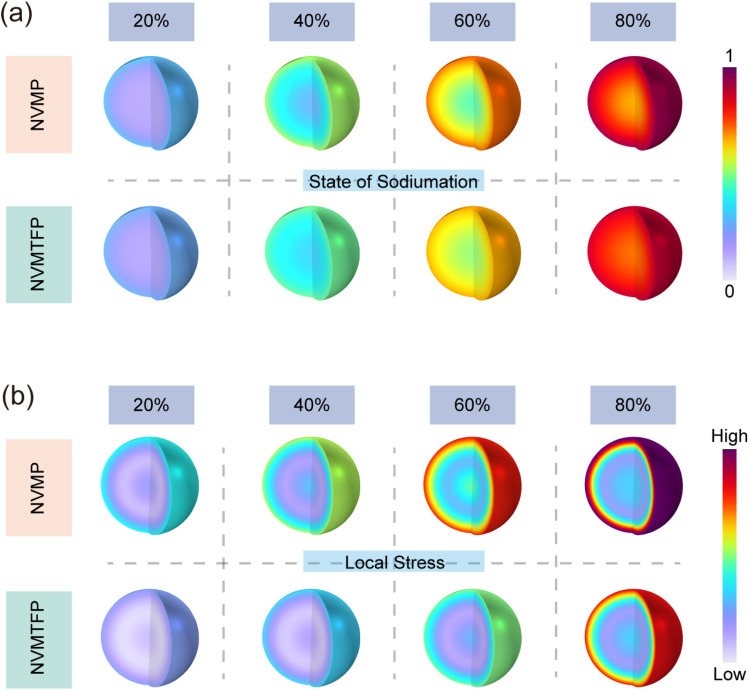
(a) Spatial distribution of Na^+^ concentration in NVMP and NVMTFP particles at different sodiation degrees. (b) Corresponding local stress distribution.

Post-cycling scanning electron microscopy (SEM) and TEM characterization further validate the positive effect of the solid-solution reaction on structural stability. Fig. S17 shows that the NVMP electrode suffers from severe cracking after cycling. Such morphological deterioration stems from volume stress accumulation induced by stepwise desodiation and localized stress concentrations during phase transitions, which ultimately induces electrode structural degradation. In contrast, NVMTFP retains an intact bulk morphology without obvious cracks. The solid-solution structure formed by Ti/Fe co-doping exerts an elastic buffering effect that effectively alleviates volume variation stress, prevents mechanical damage to particles during cycling, and safeguards electrode macrostructural integrity.^51^ TEM characterization further confirms the interfacial regulation efficacy of Ti/Fe co-doping (Fig. S18a). The cathode electrolyte interphase (CEI) layer formed by NVMP is highly heterogeneous, exhibiting localized thickening and detachment. This thick and uneven CEI layer not only increases interfacial charge transfer resistance but also fails to adequately protect the electrode, thereby accelerating structural degradation. In contrast, NVMTFP is covered by a continuous, dense, and uniform CEI layer. The uniform and thin CEI layer effectively restricts excessive electrode–electrolyte contact, suppresses side reactions, reduces charge transfer resistance, and ensures stable electrochemical kinetics.^52^ The HRTEM images after cycling are shown in Fig. S18b. For NVMP, mixed dislocations are observed, which indicates that the electrode material undergoes severe lattice distortion during cycling. These structural defects not only reduce the shear strength and ductility of the material, but also undermine the stability of the structural framework, ultimately resulting in a significant degradation of its electrochemical performance. In contrast, NVMTFP exhibits continuous and well-aligned lattice fringes with a substantially reduced dislocation density compared with NVMP, which significantly mitigates lattice distortion, preserves structural integrity, and enables better accommodation of volume changes during cycling. GPA was employed to map the stress distribution in the two electrode materials, facilitating further investigation of the observed discrepancies. As shown in Fig. S18c, NVMP exhibits significant inhomogeneity in stress distribution after cycling. In contrast, the stress distribution of NVMTFP is more uniform and homogeneous, indicating that uniformly dispersed dopant atoms in the solid solution structure establish continuous ion transport pathways, reducing structural hindrance to ion migration and thereby suppressing local stress accumulation arising from uneven ion transport. This is further corroborated by finite element COMSOL simulations, which verify stress accumulation during sodiation. Fig. 5a comparatively depicts the spatial evolution behavior during the process of sodiation for both the NVMP and the NVMTFP cathodes. As the sodiation degree progressively increases from 20% to 80%, both materials exhibit a uniform spatial elevation in their state-of-charge, indicating continuous solid-solution reaction characteristics throughout the de-/sodiation process without severe phase separation. However, Fig. 5b reveals fundamental differences in their stress evolution behavior. For NVMP, significant stress distribution heterogeneity emerges internally even at low sodiation degrees. With advancing sodiation, its overall stress level escalates sharply, accompanied by pronounced localized stress concentrations. This suggests that stepwise redox reactions induce non-uniform volume changes and accumulated phase boundaries. In stark contrast to this, NVMTFP demonstrates highly homogeneous stress distribution across the entire sodiation range. Its overall stress magnitude remains significantly lower than that of NVMP, with no observable regions of concentrated stress. These results unambiguously demonstrate that the orbital hybridization-mediated Ti/Fe synergistic doping strategy successfully transforms the originally stepwise, asynchronous redox processes in NVMP into a highly coordinated single-phase solid-solution reaction. This mechanistic shift enables volume variations induced by de-/sodiation to be uniformly distributed at the atomic scale, thereby substantially reducing local lattice stress and strain energy accumulation. Such enhanced intrinsic structural stability is of paramount importance for mitigating mechanical degradation, suppressing crack initiation and propagation during prolonged cycling, and ultimately ensuring exceptional cycle durability of the electrode material.

**Fig. 6:**
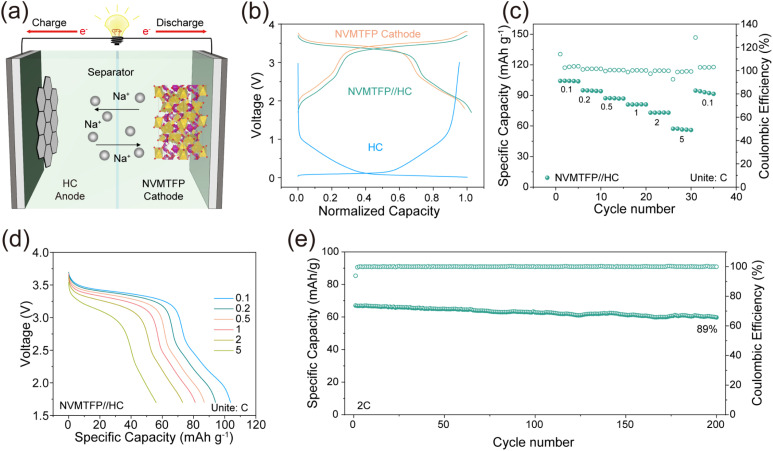
(a) Schematic illustration of the NVMTFP//HC full cell. (b) GCD curves of the NVMTFP cathode (orange), the HC anode (blue), and the assembled full cell (green). (c) Rate capability and (d) discharge curves at different rates. (e) Cycling performance of the NVMTFP//HC full cell at 2C.

To evaluate the practical application of NVMTFP, the full cells were assembled using NVMTFP as the cathode and hard carbon (HC) as the anode, denoted as NVMTFP//HC. The Na^+^ storage mechanism of the NVMTFP//HC full cell is shown in Fig. 6a. The normalized GCD curves of the NVMTFP cathode, HC anode, and NVMTFP//HC full cell are presented in Fig. 6b. The full cell exhibits a trend essentially consistent with that of the cathode, with a slight reduction in the plateau voltage attributed to the higher potential of HC relative to metallic sodium. The rate capability of the full cell, as presented in Fig. 6c, demonstrates excellent performance across various current densities. A specific capacity of 104.22 mAh g^−1^ can be achieved at 0.1C, and 61.88 mAh g^−1^ is retained at 5C. As depicted in Fig. 6d, the corresponding GCD curves exhibit notably lower polarization. Furthermore, Fig. 6e depicts the cycling performance of the NVMTFP//HC full cell at 2C. After 200 cycles, the capacity retention of the battery still reaches 89%. The excellent performance of the full cell fully verifies the effectiveness of the orbital hybridization-mediated strategy in enhancing the performance of SIBs. It should be noted that the full cell exhibits poorer rate capability and cycling stability than the half-cell, which can be attributed to the additional kinetic limitations and the continuous evolution of the SEI on the HC anode, leading to increased interfacial resistance and polarization during cycling.^53,54^ Similar behavior has also been reported in other SIB cathode systems.^55,56^

## Conclusions

In summary, this study addresses the intrinsic limitations of NVMP cathodes by developing an orbital hybridization-mediated strategy *via* Ti/Fe synergistic co-doping. The introduced Ti and Fe ions synergistically tailor the local coordination environment of the host structure. This critical modulation transforms the originally stepwise V^3+^/V^4+^ and Mn^2+^/Mn^3+^ redox reactions into a simultaneous process, fundamentally overcoming the kinetic bottleneck of the second desodiation step. Moreover, Ti/Fe co-doping constructs a continuous 3d–3d metallic network, inducing multi-orbital hybridization between transition metals and oxygen, which significantly enhances electron delocalization and intrinsic conductivity, enabling highly reversible multi-electron transfer. Systematic experimental analysis and DFT calculations validate the effectiveness of this strategy and confirm the mitigation of kinetic limitations. Benefiting from these merits, NVMTFP delivers exceptional rate capability and superior cycling stability, retaining 73% of its initial capacity after 2000 cycles at 10C. Furthermore, at 50 °C, it retains a high-capacity retention of 81% after 500 cycles at 5C. Even at −40 °C, the capacity retention remains as high as 78% after 250 cycles at 0.5C. The orbital hybridization-mediated strategy *via* co-doping provides valuable insights for the design of high-performance cathodes in SIBs.

## Author contributions

Y.-F. Liu, J.-L. Liu, and X.-T. Wang contributed equally to this work. They were responsible for conceptualization, methodology, investigation, data curation, and writing the original draft. Y. Z., J.-Z. Guo, and H. Zhang contributed to the formal analysis, validation, and visualization of the data and reviewing and editing the manuscript. D. L. Chen, Z.-Y. G., and X.-L. Wu served as the corresponding authors and were responsible for project administration, supervision, funding acquisition, and final approval of the manuscript for submission. All authors discussed the results and commented on the manuscript.

## Conflicts of interest

There are no conflicts to declare.

## Supplementary Material

SC-017-D6SC01289B-s001

## Data Availability

The data supporting the findings of this study are available within the article and its supplementary information (SI). Supplementary information is available. See DOI: https://doi.org/10.1039/d6sc01289b.
