# Supplementary material for: Orbital hybridization-mediated synergistic multi-electron redox in a NASICON cathode unlocking solid-solution reactions for ultrafast and durable sodium storage
Source: Chem Sci. 2026 Mar 23;17(18):9285–96. doi: 10.1039/d6sc01289b (PMC13007257; doi:10.1039/d6sc01289b)
Supplement: SC-017-D6SC01289B-s001 [file SC-017-D6SC01289B-s001.pdf]

Supplementary Information (SI) for Chemical Science.
This journal is © The Royal Society of Chemistry 2026

Supporting Information

Orbital Hybridization-Mediated Synergistic Multi-Electron Redox in NASICON Cathode Unlocking Solid-Solution Reaction for Ultrafast and Durable Sodium Storage

Yi-Fei Liu†, Jin-Ling Liu†, Xiao-Tong Wang†, Yan Zhuang, Jin-Zhi Guo, Heng Zhang, Denglong Chen*, Zhen-Yi Gu* and Xing-Long Wu*

1. Experimental Section

1.1 Material Synthesis

The $Na_{3.75}V_{0.75}Mn_{0.75}Ti_{0.25}Fe_{0.25}(PO_4)_3$ (NVMFTP) material was prepared via the sol-gel method. All chemical reagents were purchased from Aladdin without further purification. 0.75 mmol of ammonium metavanadate, 0.25 of mmol iron(III) nitrate nonahydrate, 0.5 of mmol diammonium titanate tetrahydrate (50 wt%) and 0.75 mmol of manganese(II) acetate tetrahydrate were added into a flask, followed by an appropriate amount of deionized water. The mixture was thoroughly stirred at 80 °C in an oil bath to form a homogeneous solution. Subsequently, 3 mmol of citric acid was prepared as a saturated solution, then slowly added to the aforementioned solution. The mixture was stirred under reflux for 1 hour, after which 3 mmol of ammonium dihydrogen phosphate and 3.75 mmol of anhydrous sodium acetate were added. Stirring continued until a wet gel was formed. The wet gel was transferred to a blast drying oven at 120 °C for overnight drying. The dried gel was ground into powder to obtain the precursor material. Then the powder was placed in a tube furnace for pre-sintering at 350 °C for 4 hours. After pre-sintering, the sample was ground again and calcined at 700 °C for 8 hours to obtain the target product. The $Na_4VMn(PO_4)_3$ (NVMP) sample was prepared via the same process, except that no iron and titanium sources were added.

1.2 Material Characterizations

X-ray diffraction (XRD, Cu Kα, λ=1.5417 Å, Thermo Nicolet IS 5, USA) was employed to analyze the composition of the physical phases of the samples, and the specific

structural information of the crystal was acquired through structural refinement. Thermogravimetric (TG) was performed on an analyzer (TG-DSC, STA409C) in the air atmosphere. The degree of graphitization of the carbon layer was characterized by Raman spectra (LabRAM HR800). Fourier transform infrared spectroscopy (Thermo Nicolet IS 5, USA) was conducted to analyze the chemical bonds of the materials. The morphology of the materials was examined by scanning electron microscopy (SEM, Hitachi SU 8000) and transmission electron microscopy (TEM, JEOL-2100F). The element distribution of each compound was obtained by TEM equipped with an energy dispersive spectrometer (EDS). X-ray photoelectron spectroscopy (XPS) was conducted using an electron spectrometer (VG Scientific ESCALab 220 i-XL) with Al Kα radiation to determine the valence states of the elements in the materials. *In-situ* XRD patterns were collected during cycling using a custom cell that employed a beryllium plate simultaneously as the X-ray window and current collector, with galvanostatic tests conducted at 0.3 C.

1.3 Electrochemical measurements

Electrochemical performance: The cathode electrode was prepared by mixing the active material, conductive carbon black (Super C45), and sodium carboxymethyl cellulose (CMC) in distilled water at a ratio of 7:2:1, coating it on Al foil in the form of a slurry, and preparing the electrodes. The electrodes were dried in a vacuum oven at 60°C overnight. Mostly, the loading mass of active material on the electrode is at the range of 1.0~1.2 mg cm^{-2}.

Cell assembly and test: CR2032 type coin cells were assembled in a glove box with

a water and oxygen content of less than 0.1 ppm, using metal sodium as the counter and reference electrode, a glass microfiber membrane (Whatman) as the separator, and an electrolyte of 1 M $NaClO_4$ in propylene carbonate (PC) with 10 vol% fluoroethylene carbonate (FEC). It should be noted that the electrolyte employed at -40°C was 0.8 M $NaPF_6$ in a diethylene glycol dimethyl ether and 1,3-dioxolane solution with a volume ratio of 8:2. The charge-discharge tests were conducted on a battery testing system (NEWARE CT-4000). Cyclic voltammetry (CV) was tested using a CHI660 electrochemical workstation. The galvanostatic intermittent titration technique (GITT) tests were conducted at a current density of 0.1 C, with current pulses lasting for 0.5 h and resting for 4 h during each titration course. EIS tests were performed on an electrochemical workstation (Solartron 1260A). The voltage range for the half-cells test was 1.8~3.8 V. EIS tests were performed on an electrochemical workstation (Solartron 1260A). In-situ EIS tests were performed at different states of charge. The half-cells were charged using a galvanostatic current, and an EIS test was performed each time the voltage increased by 0.1 V. The test method for the discharge process was the same as the charge process. The frequency range of the EIS test was 10^{-2} Hz to 10^{6} Hz with an amplitude of 5 mV. The electronic conductivity was measured at room temperature using the four-probe direct current method on powder samples under different pressures.

The full cells were assembled using the NVMTFP cathode and HC anode, with a voltage window of 1.7~3.7 V. Prior to assembling the full cells, pre-sodiation was conducted on the HC anode to activate and stabilize its electrode surface. The hard

carbon anode was made in the same way as the cathode, except that the ratio was 8:1:1. The HC anode and sodium sheet were directly short-circuited by dropping electrolyte, followed by assembling into a short-circuited half-cell under a certain pressure. Pre-sodiation was achieved by standing for 12 hours. Then the cell was disassembled, and the HC anode and NVMTFP cathode were assembled into a full cell, with a cathode and anode electrode mass ratio of about 1.3:1.

1.4 COMSOL simulations

The stress simulation of NVMP and NVMTFP particles was conducted using the Solid Mechanics module in COMSOL Multiphysics software. The particles were geometrically simplified as ideal spheres with a diameter of 300 nm, and a constant current density of 5 A m^{-2} was applied to characterize the distribution of Na^+ within the particles.[1] By defining the chemical strain field related to sodium concentration, the internal stress evolution caused by lattice expansion during sodium desorption was simulated. The boundary conditions were set as free surface and symmetry constraints. The steady-state and transient solvers were used to calculate the von Mises stress, principal stress, and displacement distribution under different sodium contents, and the mechanical stability and fragmentation risk of the material during the cycling process were analyzed.[2]

2.Calculation Section

2.1 Calculation of diffusion kinetics

The apparent sodium ion diffusion coefficients ($D_{app,Na}$) were further calculated by GITT tests to evaluate the sodium ion diffusion kinetics of the NVMTFP or NVMP material. The $D_{app,Na}$ can be calculated by the following formula:[3]

$$D_{app,Na} = \frac{4}{\pi\tau}\left(\frac{m_B V_M}{M_B S}\right)^2\left(\frac{\Delta E_s}{\Delta E_\tau}\right)^2 \qquad (\tau \ll L^2/D)\#$$

Where τ is the duration of applying a constant current; V_M is the molar volume of the active material; M_B and m_B are the molar mass and mass of the active material, respectively; S is the electrode surface area (1.13 cm^2), and L is the particle size of the active material; $\triangle E_s$ and $\triangle E_\tau$ are the equilibrium voltage during a single titration and the total voltage change, respectively.

2.2 Computational Details

The density functional theory (DFT) calculation[4] was implemented in the Vienna Ab initio simulation package (VASP). The exchange-correlation potential is described by using the generalized gradient approximation of Perdew-Burke-Ernzerhof (GGA-PBE).[5] The projector augmented-wave (PAW) method is employed to treat interactions between ion cores and valence electrons.[6] The plane-wave cutoff energy was fixed to 450 eV. Given structural models were relaxed until the Hellmann – Feynman forces smaller than -0.02 eV/Å and the change in energy smaller than 10^{-5} eV was attained. The vacuum thickness was set to be 15 Å to minimize interlayer interactions. During the relaxation, the Brillouin zone was represented by a Γ centered k-point grid of 4×6×2.[7] Grimme's DFT-D3 methodology was used to describe the dispersion interactions among all the atoms in adsorption models.

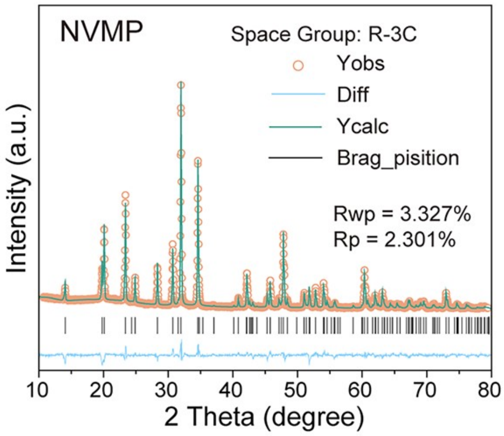

Figure S1. The XRD Rietveld refinements of NVMP.

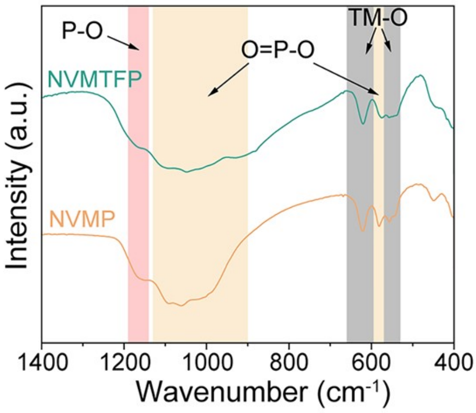

Figure S2. FT-IR spectra of NVMP and NVMTFP.

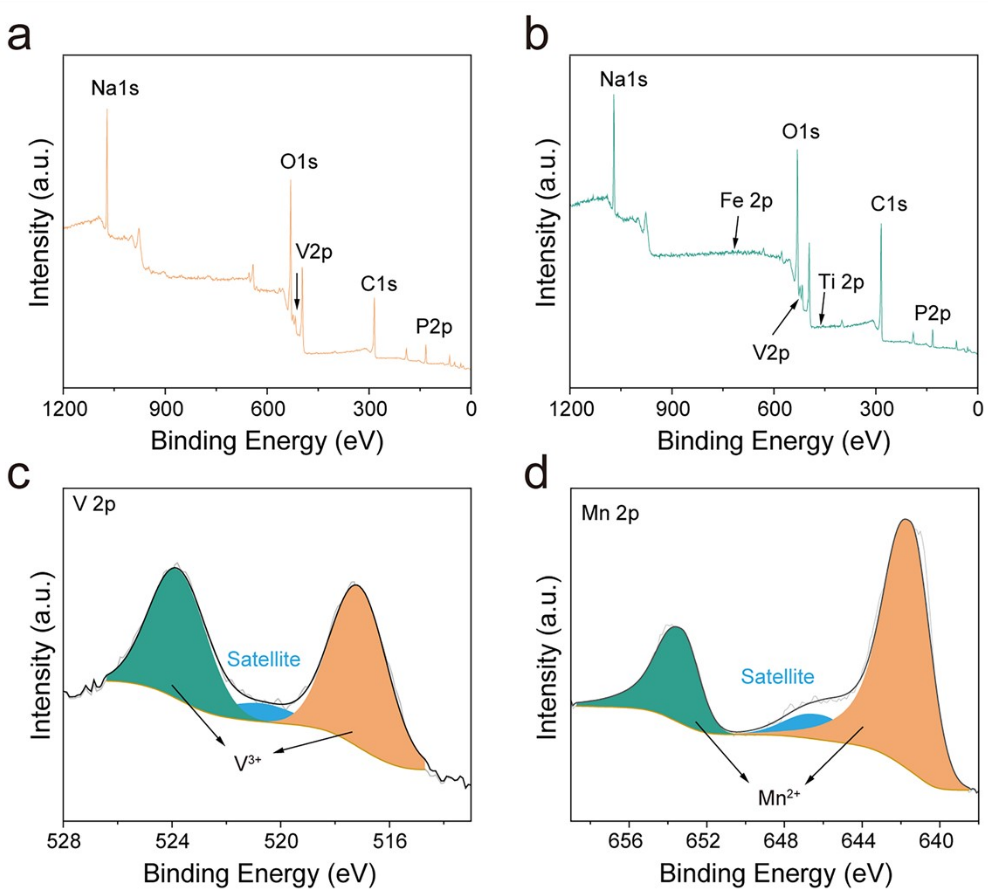

Figure S3. Full-spectrum XPS survey of (a) NVMPand (b) NVMTFP. High-resolution XPS spectrum of (c) V2p and (d) Mn 2P.

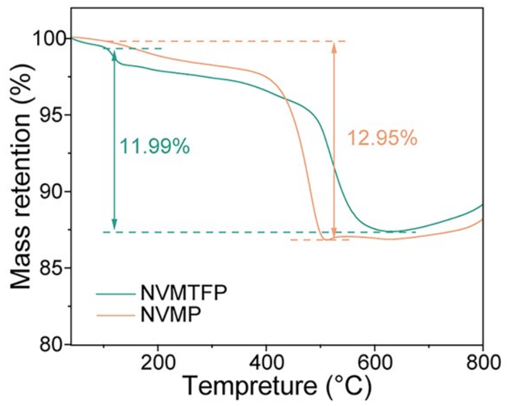

Figure S4. TGA curves of NVMP and NVMTFP materials.

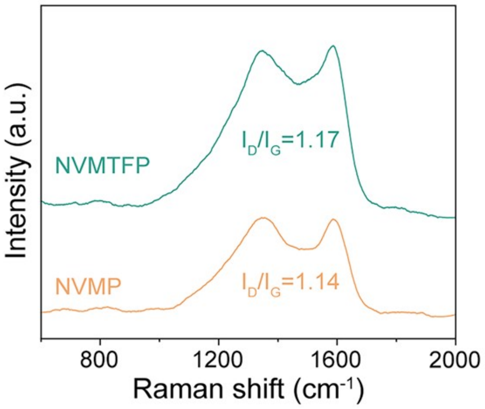

Figure S5. Raman spectra of NVMP and NVMTFP.

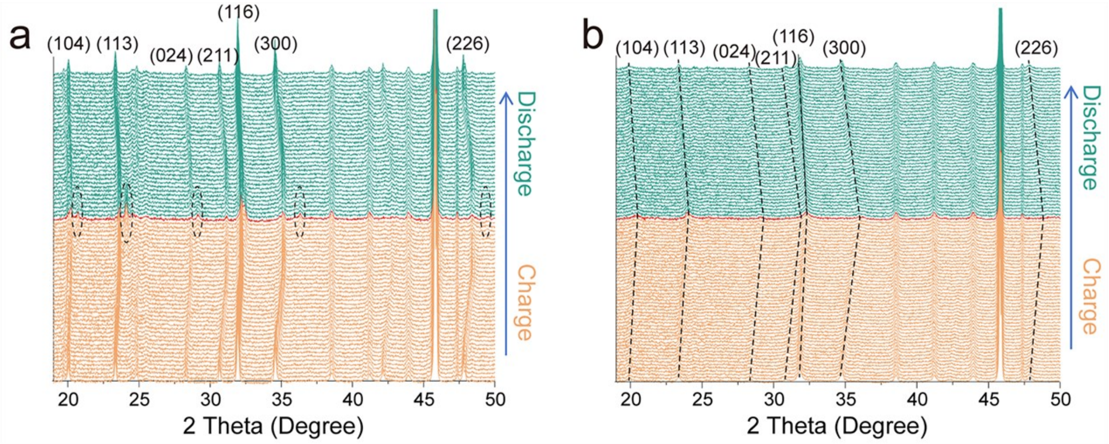

Figure S6. *In-situ* XRD patterns (19.5-50°) of (a) NVMP and (b) NVMTFP during the cycle.

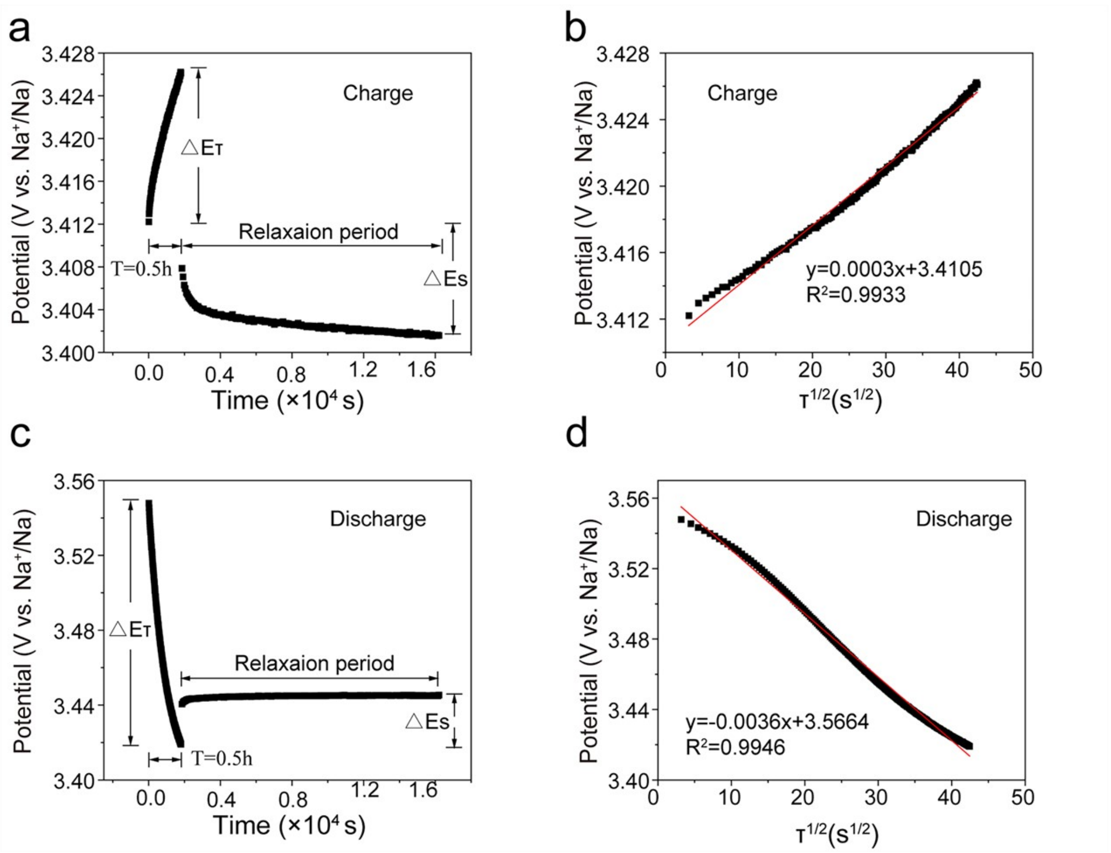

Figure S7. The relationship between *τ* and *E* during a single GITT process and the linear fitting of *E* and $\tau^{1/2}$ for the NVMP half-cell. (a) and (b) Charging process. (c) and (d) Discharging process.

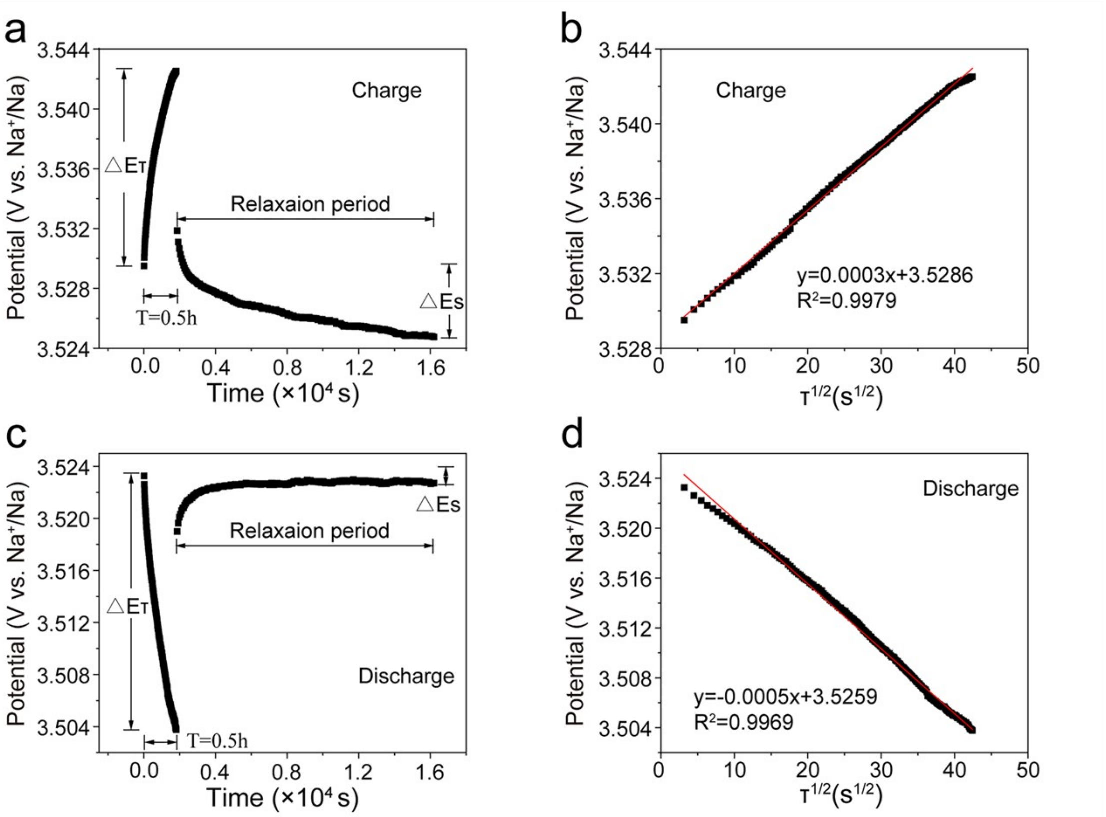

Figure S8. The relationship between *τ* and *E* during a single GITT process and the linear fitting of *E* and $\tau^{1/2}$ for the NVMTFP half-cell. (a) and (b) Charging process. (c) and (d) Discharging process.

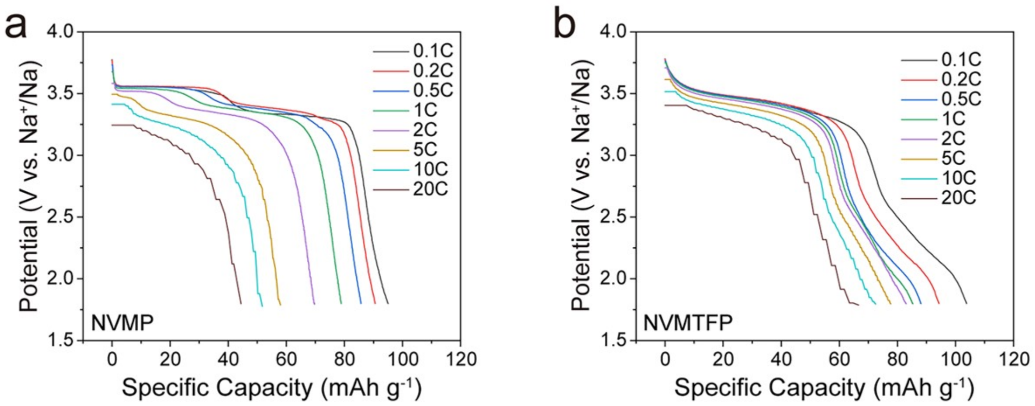

Figure S9. The discharge curves of (a) NVMP and (b) NVMTFP cathodes at different rates.

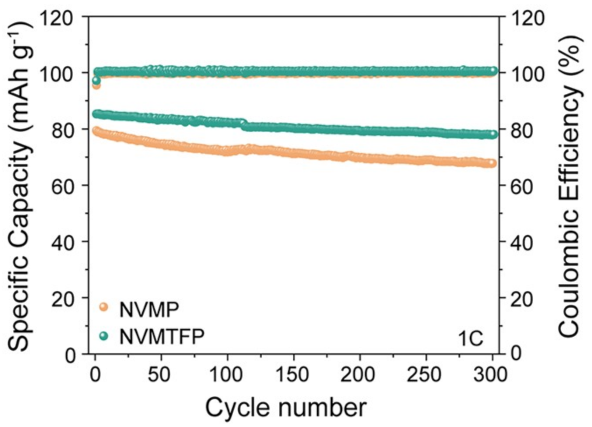

Figure S10. Cycling performance of NVMP and NVMTFP at 1C.

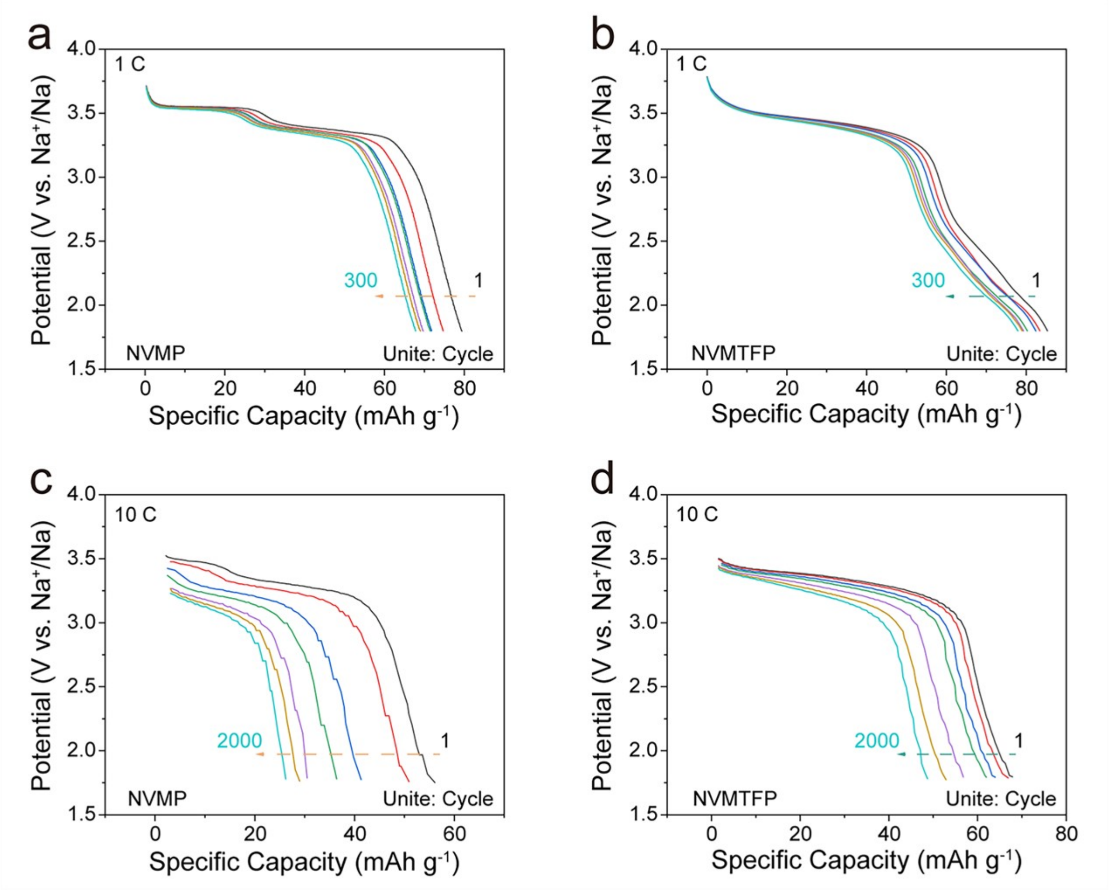

Figure S11. The discharge curves of (a) NVMP and (b) NVMTFP cathodes at 1 C, and the discharge curves of (c) NVMP and (d) NVMTFP cathodes at 10 C.

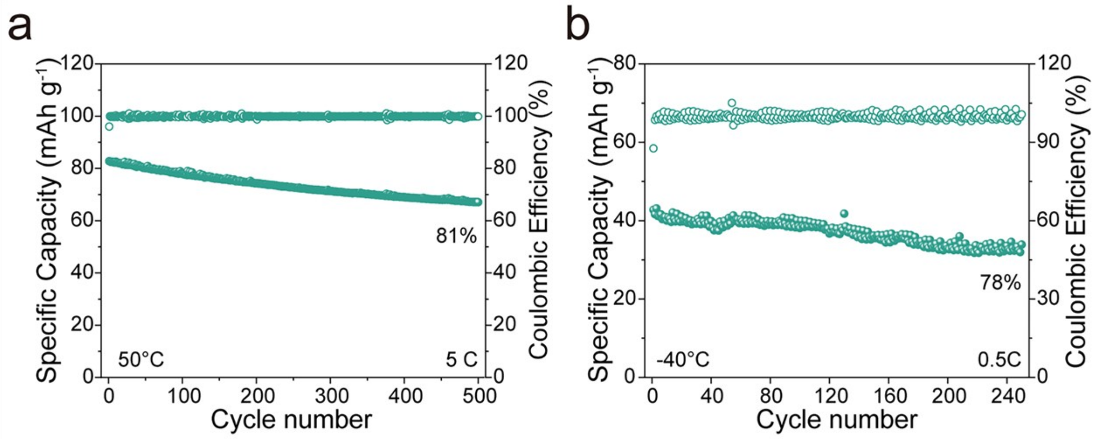

Figure S12. Cycling performance at different temperatures of NVMTFP.

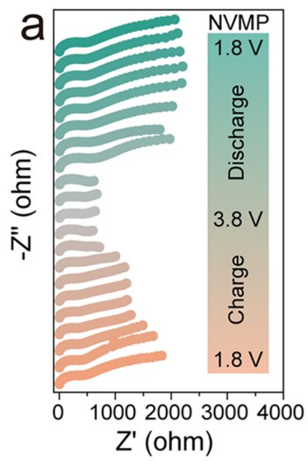

Figure S13. The in situ EIS Nyquist plots of (a) NVMP and (b) NVMTFP.

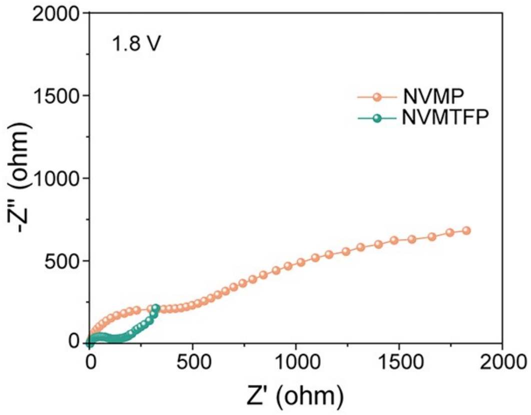

Figure S14. Nyquist plots of the NVMP and NVMTFP at 1.8 V.

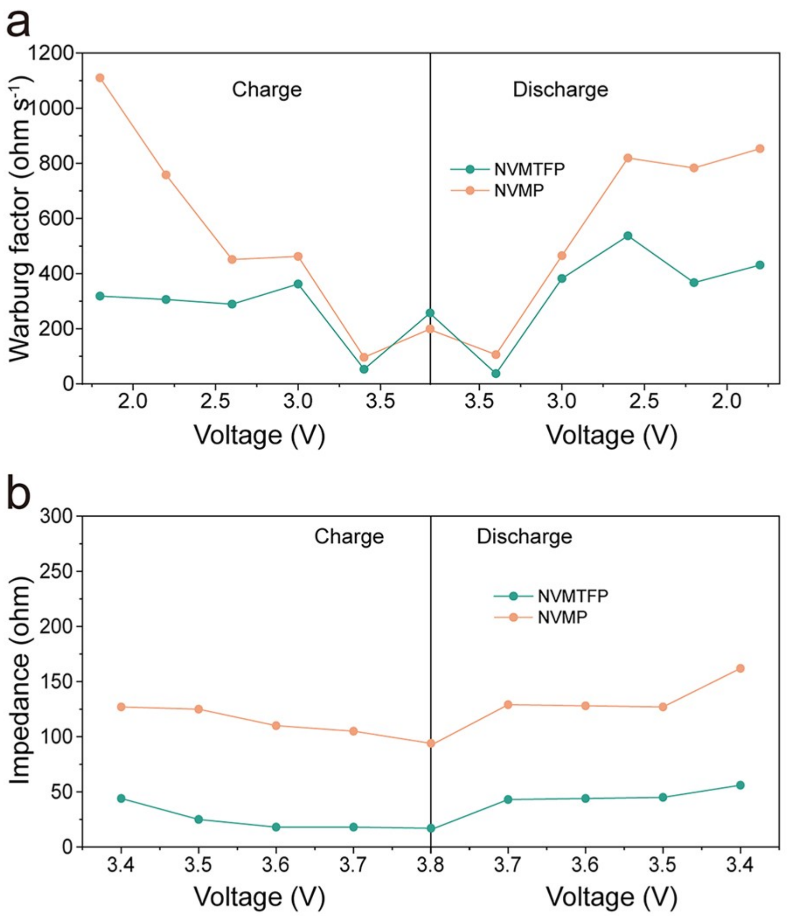

Figure S15. The variation of (a) diffusion impedance and (b) charge transfer impedance during the charging and discharging processes.

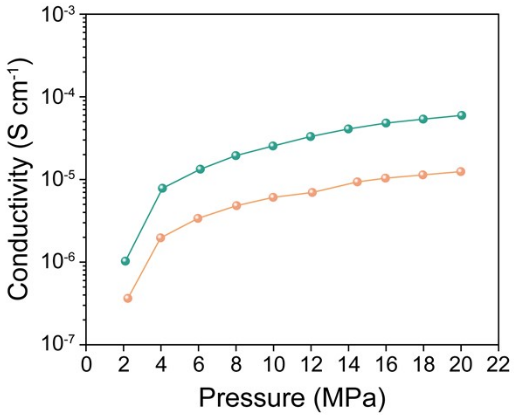

Figure S16. Pressure dependence of conductivity of NVMP and NVMTFP.

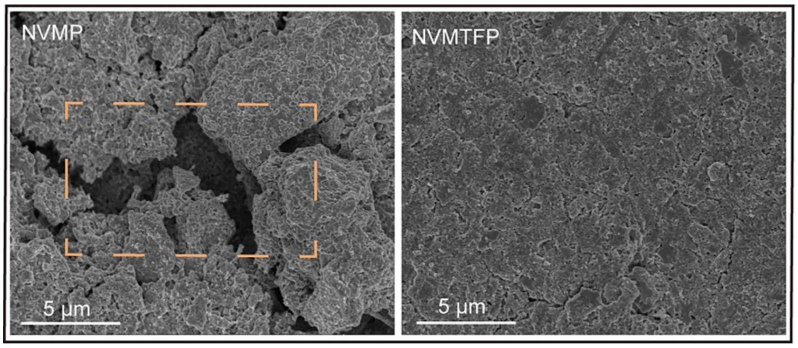

Figure S17. SEM images of NVMP and NVMTFP after cycling.

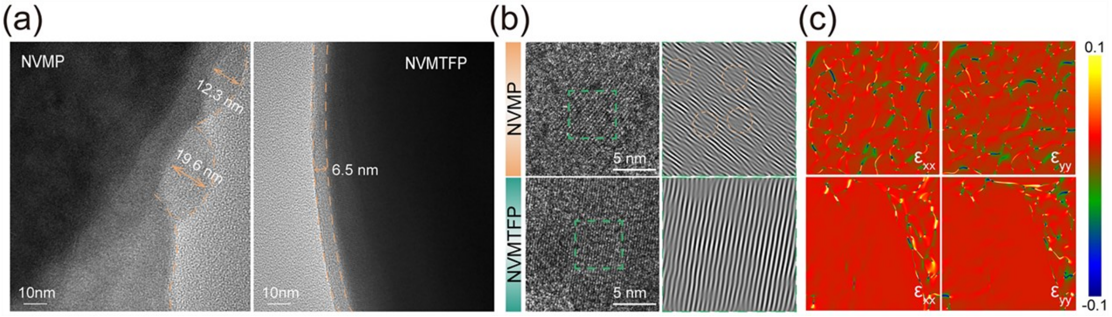

Figure S18. (a) TEM images of NVMP and NVMTFP after cycling. (b) HRTEM and dislocation distribution images. (c) Corresponding localized strain distribution analysis by GPA.

Table S1. Lattice parameters of NVMP and NVMTFP obtained from XRD Rietveld refinements.

Samples	a(b)/Å	c/Å	Volume/Å^3
NVMP	8.9760	21.4629	1497.57
NVMTFP	8.8560	21.7066	1474.32

Table S2. Refined structural parameters of the NVMP.

Atom	Type	Site	x	y	z	Occ.
Na	Na1	6b	0.33333	0.66667	0.16667	1
Na	Na2	18e	0.66667	0.9686	0.08333	1
V	V1	12c	0.33333	0.66667	0.01775	0.5
Mn	Mn	12c	0.33333	0.66667	0.01775	0.5
P	P1	18e	-0.0319	0.33333	0.08333	1
O	O1	36f	0.14193	0.49765	0.07762	1
O	O2	36f	0.54047	0.8448	0.02643	1

Table S3. Refined structural parameters of the NVMTFP.

Atom	Type	Site	x	y	z	Occ.
Na	Na1	6b	0.33333	0.66667	0.16667	1
Na	Na2	18e	0.66667	0.9697	0.08333	1
Mn	Mn1	12c	0.33333	0.66667	0.01727	0.375
Ti	Ti1	12c	0.33333	0.66667	0.01727	0.125
V	V1	12c	0.33333	0.66667	0.01727	0.375

Fe	Fe1	12c	0.33333	0.66667	0.01727	0.125
P	P1	18e	-0.0355	0.33333	0.08333	1
O	O1	36f	0.14193	0.49765	0.07762	1
O	O2	36f	0.54047	0.8448	0.02643	1

Reference

1. Y. Xiao, Q. Sun, D. Chen, J. Wang, J. Ding, P. Tan, Y. Sun, S. Zhang, P. Wang, J. Mao and Y. Zhu, Guideline of Dynamic Tunnel Structural Evolution for Durable Sodium-Ion Oxide Cathodes, Adv. Mater., 2025, 37, 2504312.
2. T. Zhang, M. Sotoudeh, X. Yao, A. Groß and M. Kamlah, 3D chemo-mechanical modeling of microstructure evolution and anisotropic deformation in $Na_xV_2(PO_4)_3$ cathode particles for sodium-ion batteries, Int. J. Solids Struct., 2025, 320, 113525.
3. H. Li, M. Xu, C. Gao, W. Zhang, Z. Zhang, Y. Lai and L. Jiao, Highly efficient, fast and reversible multi-electron reaction of $Na_3MnTi(PO_4)_3$ cathode for sodium-ion batteries, Energy Storage Mater., 2020, 26, 325-333.
4. W. Kohn and L. Sham, Self-Consistent Equations Including Exchange and Correlation Effects, Phys. Rev., 1965, 140, A1133-A1138.
5. J. Perdew, K. Burke and M. Ernzerhof, Generalized Gradient Approximation Made Simple, Phys. Rev. Lett., 1996, 77, 3865-3868.
6. P. Blöchl, Projector augmented-wave method, Phys. Rev. B, 1994, 50, 17953-17979.
7. H. Monkhorst and J. Pack, Special points for Brillouin-zone integrations, Phys. Rev. B, 1976, 13, 5188-5192.
